# Nutritional treatment with an immune-modulating enteral formula alleviates 5-fluorouracil-induced adverse effects in rats

**DOI:** 10.1371/journal.pone.0225389

**Published:** 2019-11-26

**Authors:** Kentaro Nakamura, Hidekazu Tonouchi, Akina Sasayama, Taketo Yamaji, Kinya Ashida

**Affiliations:** Nutrition Research Department, Food Microbiology & Function Research Laboratories, R&D Division, Meiji Co., Ltd., Hachiouji, Tokyo, Japan; University of Illinois, UNITED STATES

## Abstract

Cancer chemotherapy is frequently accompanied by adverse effects, such as diarrhoea and leukopenia, which lead to malnutrition and a decrease in the patients’ quality of life. We previously demonstrated that an immune-modulating formula (IMF)—an enteral formula enriched with immunonutrients, whey-hydrolysed peptides, and fermented milk—had anti-inflammatory effects and protective effects on intestinal disorders in some experimental models. Here, we investigated whether nutritional treatment with the IMF could prevent 5-fluorouracil (5-FU)-induced adverse effects in rats. Rats were randomised into CTR and IMF groups, which received a control formula or the IMD supplemented formula *ad libitum*. Two weeks after starting the formula, rats were intraperitoneally injected with 5-FU (300 mg/kg) on day 0. The treatment with 5-FU decreased their body weights, food intake, and leukocyte counts, and worsened the diarrhoea score. However, the body weights, food intake, and leukocyte counts were significantly higher in the IMF rats than in the CTR rats on day 1. The IMF also delayed the incidence of diarrhoea and significantly preserved the villus heights in the jejunum on day 2. In conclusion, nutritional treatment with the IMF alleviated the adverse effects induced by 5-FU injection in rats.

## Introduction

The adverse effects of chemotherapeutic agents, such as gastrointestinal toxicity and leukopenia, often lead to a reduction of the dosage, discontinuation of cancer treatment, malnutrition, or a reduction in the patients’ quality of life. Moreover, malnutrition in cancer patients worsens treatment outcome, incidence of chemotherapy toxicity and quality of life [1, 2]. It is important to continue cancer chemotherapy, while maintaining patients’ nutritional status and without (or while controlling) the occurrence of adverse effects. 5-fluorouracil (5-FU) is one of the most commonly used chemotherapeutic agents for various cancer treatments [3]. Like any other chemotherapy agent, 5-FU has many adverse effects, such as diarrhoea, gastrointestinal mucositis, poor appetite, leukopenia, and nausea. In relation to gastrointestinal mucositis, the injection of 5-FU has been shown to induce pro-inflammatory cytokines and NF-kappaB activation in the small intestine [4, 5]. An anti-inflammatory drug (5-aminosalicylic acid) has been shown to improve 5-FU-induced intestinal injury and to inhibit NF-kappaB activation and pro-inflammatory cytokine production [4].

It has been reported that the adverse effects of chemotherapeutic agents compromise patients’ quality of life (QOL), whereas complementary and alternative medicines improve patients’ QOL [6]. Nutritional modulation has also been reported to benefit cancer patients during chemotherapy [7]. Recently, certain nutrients, classified as immunonutrients, such as specific amino acids, fatty acids, and vitamins have been shown to modulate the immune system. There is also an immune-modulating formula (IMF), which is an enteral nutritional formula intended for specific dietary uses and which is enriched with nutrients having anti-inflammatory properties [8]. An IMF enriched with whey-hydrolysed peptides, fermented milk, omega-3 polyunsaturated fatty acids, and anti-oxidant molecules (vitamin A, C, E, zinc, and selenium) has been demonstrated to protect the small intestine against indomethacin-induced gastrointestinal disorders [9] as well as to have anti-inflammatory effects [10–13].

Here, to clarify the benefits of nutritional treatment with the IMF during the chemotherapy, we investigated whether the IMF could prevent 5-fluorouracil-induced adverse effects in rats.

## Materials and methods

### Ethics statement

All animal experiments reported herein were approved by the Ethics Committee for Animal Care and Use of Meiji Co., Ltd. (Tokyo, Japan) (approval #2012_3871_0093/0094, approval date 3 Sept 2012, and approval #2013_3871_0009/0014, approval date 9 Apr 2013). The experiments were carried out from September 2012 to July 2013 in strict accordance with the guidelines of this committee, which were based on Guide for the Care and Use of Laboratory Animals (National Research Council Japan). All surgeries were performed under deep anaesthesia with isoflurane, and all efforts were made to minimise animal suffering. When symptoms such as severe body weight loss and hunching behaviour were observed before the end of the experiment, the rats were euthanised with carbon dioxide gas.

### Animals

Six-week-old male Wistar rats were purchased from Japan SLC (Hamamatsu, Japan). The rats were housed in wire-bottom cages under controlled temperature and humidity with a 12-h light/dark cycle and fed commercial feed with water *ad libitum* for 1 week prior to use in the experiments.

### Chemicals and diets

5-FU (5-FU injection, 250 mg 5-FU in 5 mL solution, Kyowa Hakko Kirin Co, Ltd, Japan) was purchased from Wako Pure Chemical Industries (Osaka, Japan). The control enteral formula (Meibalance HP; Meiji Co., Ltd.) and the immune-modulating enteral formula (MHN-02; Meiji Co., Ltd.) were purchased from Meiji Co., Ltd. in the liquid form. Compositions of the formulas are listed in Table 1. The control enteral formula and the IMF were purchased sterile, wherein the lactic acid bacteria in the fermented milk were heat killed. These formulas were lyophilised, and then vacuum-packed and refrigerated with an oxygen absorber and desiccants until administration to avoid rotting and oxidation.

**Table 1:** Nutritional contents of the test formulas (per 100 kcal).

	Control enteral formula	Test formula (50% of control/50% of IMF)
Protein (g)	5.0	5.0
Protein sources	Milk protein, Sodium caseinate	Whey-hydrolysed peptides, fermented milk, Milk protein, Sodium caseinate
Carbohydrates (g)	15.3	14.9
Carbohydrate sources	Dextrin	Dextrin, Isomaltulose
Lipids (g)	2.5	2.65
Lipid sources	LCT ^a^	LCT, MCT ^b^, EPA ^c^, DHA ^d^
*Vitamins*		
Vitamin A (μg RE ^e^)	60	105
Vitamin D (μg)	0.50	0.63
Vitamin E (mg)	3.0	4.0
Vitamin K (μg)	3.1	3.3
Vitamin B1 (mg)	0.15	0.2
Vitamin B2 (mg)	0.20	0.25
Niacin (mg)	1.6	2.3
Vitamin B6 (mg)	0.30	0.30
Vitamin B12 (μg)	0.60	0.60
Folic acid (μg)	50	50
Biotin (μg)	15.0	11.3
Vitamin C (mg)	16	33
Choline (mg)	1.7	5.5
*Minerals*		
Sodium (mg)	110	90
Potassium (mg)	100	90
Calcium (mg)	60	70
Magnesium (mg)	20	20
Phosphorus (mg)	60	65
Iron (mg)	1.0	1.0
Zinc (mg)	0.8	0.9
Copper (mg)	0.080	0.065
Manganese (mg)	0.20	0.19
Chromium (μg)	3.0	3.0
Molybdenum (μg)	2.5	2.5
Selenium (μg)	3.5	4.3
Iodine (μg)	15	12.4
Chloride (mg)	140	110

^a^LCT, long chain triglycerides

^b^MCT, medium chain triglycerides

^c^EPA, eicosapentaenoic acid

^d^DHA, docosahexaenoic acid

^e^RE, retinol equivalent

### Investigation on the incidence of adverse effects

Forty rats were randomised into Control (CTR; n = 20) and IMF (n = 20) groups. The control group received the control enteral formula (Meibalance HP, powder form), and the IMF group received the test formula, in which 50% of the control enteral formula was substituted with the IMF (MHN-02, powder form), *ad libitum*.

Two weeks after starting the respective formulas, rats were weighed and administered a single dose of 5-FU (300 mg/kg body weight) by intraperitoneal injection on day 0.

Body weight and food intake were recorded daily and diarrhoea was scored twice a day until day 4. Blood samples were collected daily by lateral tail vein and assayed for haematological analysis. All rats were euthanised by bleeding from the abdominal aorta under deep anaesthesia with isoflurane at the end of the experiment.

### Haematological analysis

Whole blood samples were treated with EDTA. Total and differential leukocyte counts and counts of lymphocytes, neutrophils, and monocytes were measured using an automatic haematology analyser (XT-1800i; Sysmex, Hyogo, Japan).

### Diarrhoea assessment

Diarrhoea was scored twice a day until day 4 according to a scale described in previous studies [14, 15]: 0 (normal; normal stool or absent); 1 (slight; slightly wet and soft stool); 2 (moderate; wet and unformed stool with moderate perianal staining of the coat); 3 (severe; watery stool with severe perianal staining of the coat).

### Histological analysis

Control (CTR; n = 20) and IMF (n = 20) rats, which received the control enteral formula and the test formula respectively *ad libitum*, were euthanised by bleeding from the abdominal aorta under deep anaesthesia with isoflurane two days after the administration of 5-FU, and their proximal and distal small intestines (jejunum and ileum, respectively) were collected and fixed in 10% buffered formalin. Formalin-fixed specimens were processed and embedded in paraffin. From these specimens, 3-micrometer paraffin sections were stained with haematoxylin-eosin staining and photographed using a digital microscope (Keyence, Osaka, Japan). Mean villus height and crypt depth measurements were obtained by evaluating 40 villi and crypts per rat.

### Statistical analysis

Data are presented as means ± standard deviations. Comparisons between two groups were performed using the Shapiro–Wilks test for normality and the F-test for variance, followed by Student’s *t*-test for homoscedastic data or Aspin–Welch’s *t*-test, since the data were normally distributed. The Mann–Whitney *U*-test was used for data not normally distributed. Differences were considered significant at *P* < 0.05.

## Results

### Body weight and food intake

**Fig 1:**
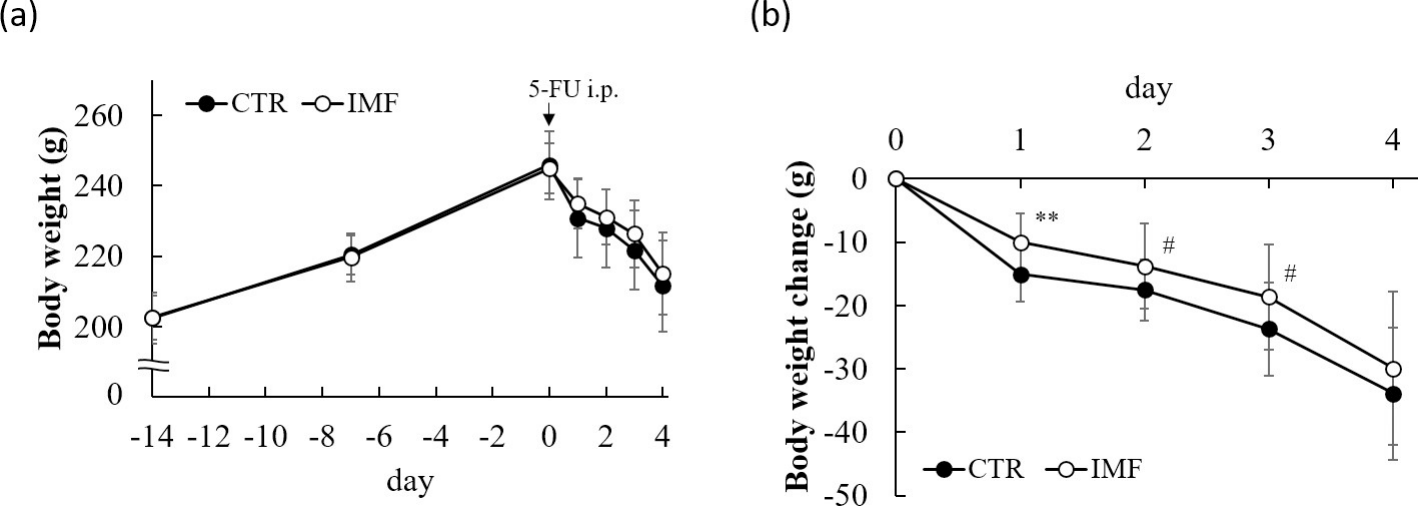
Body weight change over time in the CTR and IMF rats. (**a**) body weight and (**b**) body weight change from day 0. Values are means ± standard deviations. # *P* < 0.1, ** *P* < 0.01, vs. CTR group.

No significant differences in the body weight and food intake were observed between the groups before 5-FU injection (Fig 1A and Table 2). Treatment with 5-FU reduced the body weight and food intake in both groups. However, the reduction of body weight was significantly lower and food intake was significantly higher in the IMF rats than in the CTR rats on day 1 (Fig 1B and Table 2). The body weight in the IMF rats tended to decrease compared with that in the CTR rats on days 2 and 3 (Fig 1B).

**Table 2:** Food intake after 5-FU injection.

Day	CTR (g/day)	IMF (g/day)
~ Day 0	13.6 ± 0.8	13.8 ± 0.8
Day 0 ~ Day 1	3.2 ± 1.1	4.6 ± 2.0 **
Day 1 ~ Day 2	7.2 ± 2.7	7.7 ± 2.1
Day 2 ~ Day 3	4.5 ± 4.9	5.2 ± 2.7
Day 3 ~ Day 4	2.8 ± 4.7	2.6 ± 3.6

Values are mean ± standard deviations.

** *P* < 0.01, vs. CTR group by Student’s *t*-test.

### Leukocyte counts

**Fig 2:**
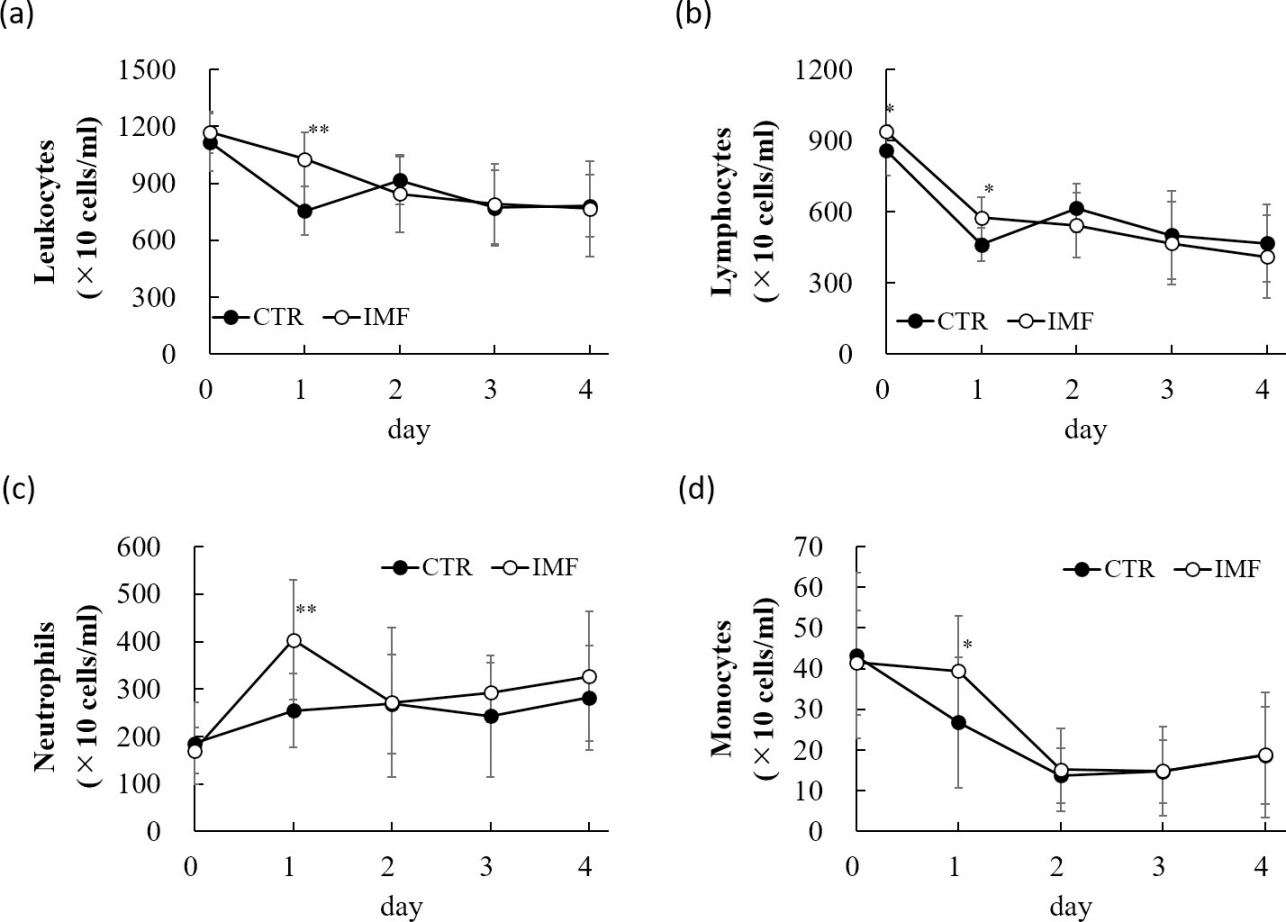
The counts of total leukocytes, lymphocytes, neutrophils, and monocytes after 5-FU injection. (**a**) total leukocyte counts, (**b**) lymphocyte count, (c) neutrophil count, and (d) monocyte count. Values are means ± standard deviations. * *P* < 0.05, ** *P* < 0.01, vs. CTR group.

Total leukocyte counts, and the counts of neutrophils and monocytes were not significantly different between the two groups before the 5-FU injection (day 0) (Fig 2A, 2B and 2C). Lymphocyte count was significantly higher in the IMF group than in the CTR group at day 0 (Fig 2B). The 5-FU treatment reduced the leukocyte, lymphocyte, and monocyte counts, and increased the neutrophil count. However, the IMF rats significantly preserved their leukocytes, lymphocytes, and monocytes compared with those of the CTR rats on day 1 (Fig 2A, 2B and 2D). The count of the neutrophils was significantly higher in the IMF rats than in the CTR rats on day 1 (Fig 2C).

### Diarrhoea and histological analysis

**Fig 3:**
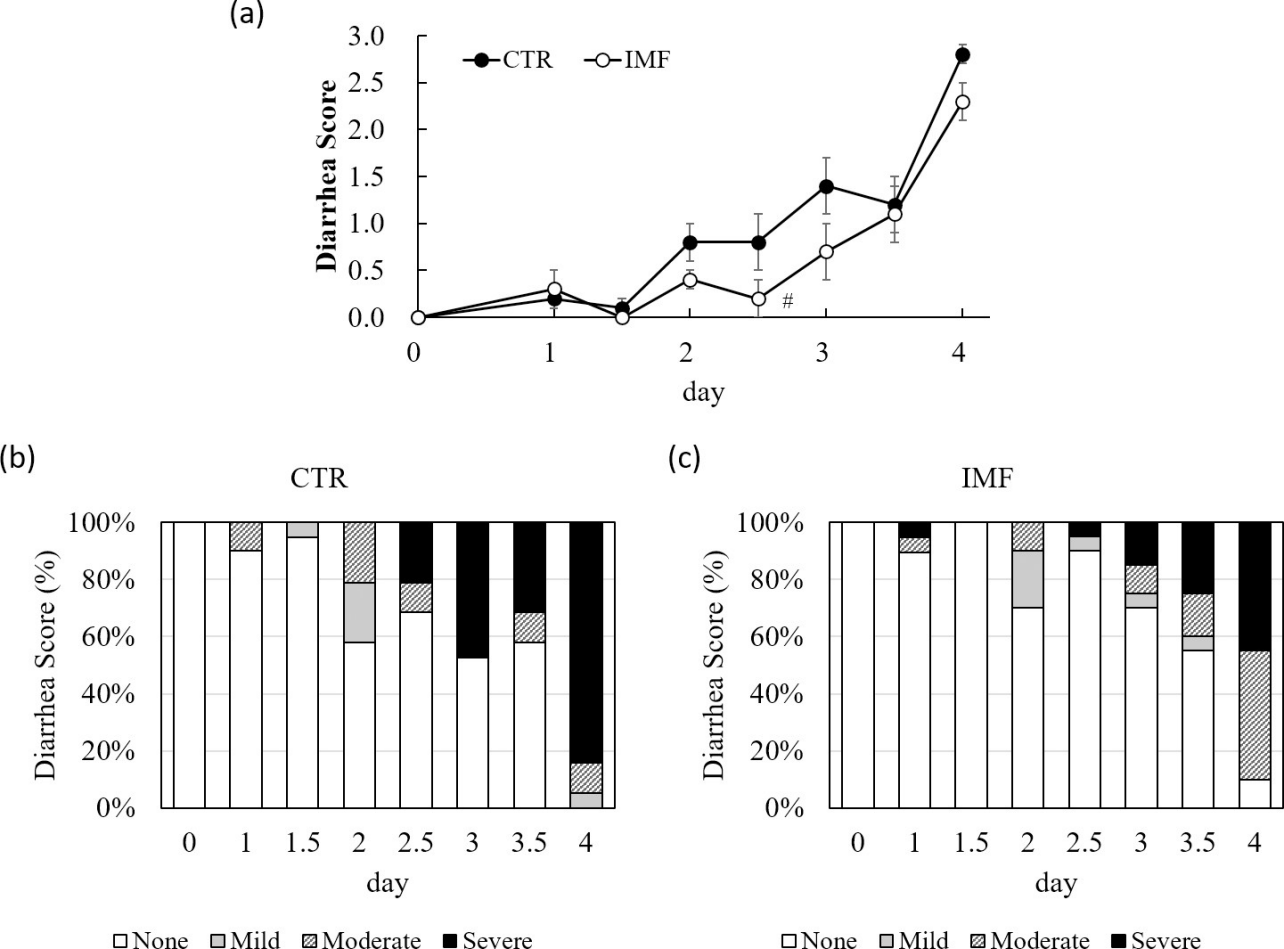
Average diarrhoea score and incidence of diarrhoea over time in the CTR and IMF rats. (**a**) average diarrhoea score, (**b**) incidence of diarrhoea in the CTR rats after 5-FU injection and (**c**) incidence of diarrhoea in the IMF rats after 5-FU injection. Values are means ± standard error. # *P* < 0.1, vs. CTR group.

Although the diarrhoea score was worsened in both groups after 5-FU injection, the average diarrhoea score in the CTR rats was higher than that in the IMF rats throughout the experiment (Fig 3A). The incidence of diarrhoea was delayed in the IMF rats (Fig 3B and 3C).

**Fig 4:**
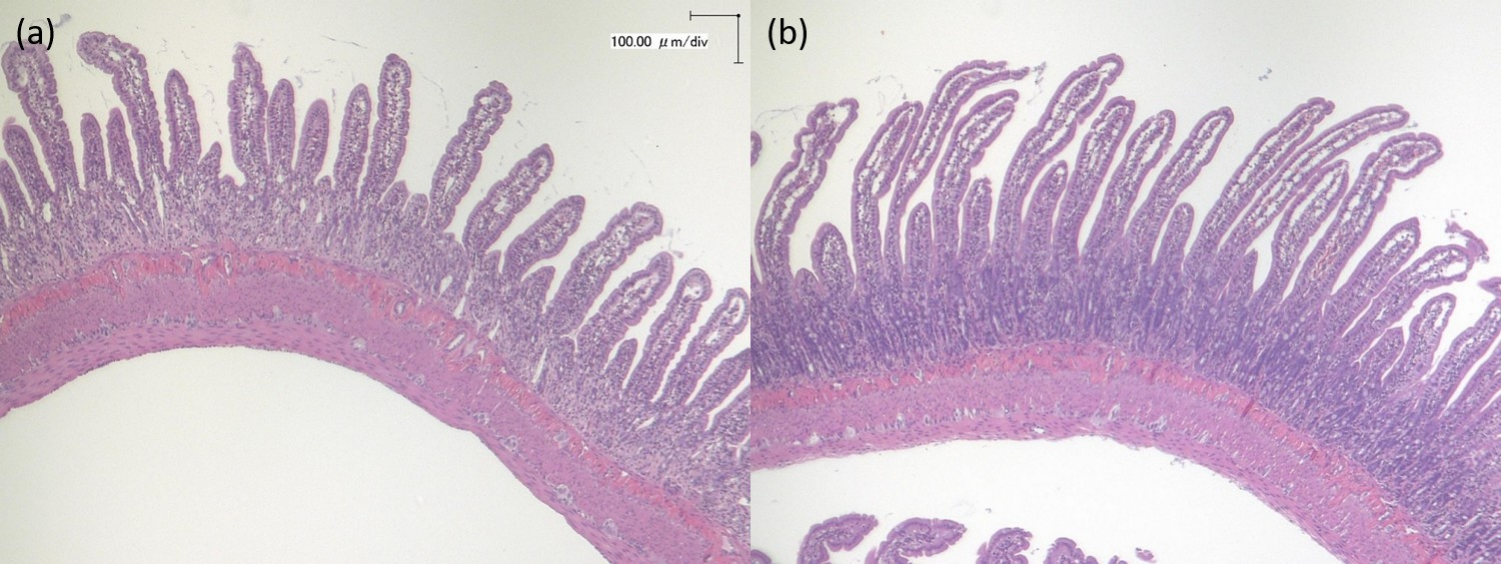
Microscopic features of the jejunum. (**a**) CTR group and (**b**) IMF group.

We previously observed that 5-FU administration caused mucosal damage in the small intestine, which was associated with the incidence of diarrhoea. The IMF rats showed significantly greater villus height and mucosal layer thickness in the jejunum than the CTR rats on day 2 (Table 3 and Fig 4).

**Table 3:** The morphometry of intestinal villus height, crypt depth, lamina muscularis mucosa, mucosal layer thickness, and muscle layer thickness.

	CTR	IMF
Villus height (μm)	353.2 ± 50.6	394.0 ± 37.2 **
Crypt depth (μm)	114.8 ± 22.7	118.5 ± 16.6
Lamina muscularis mucosa (μm)	32.7 ± 4.4	31.2 ± 3.7
mucosal layer thickness (μm)	500.7 ± 60.1	543.7 ± 48.4 *
Muscle layer thickness (μm)	103.8 ± 17.3	109.8 ± 14.2

Values are mean ± standard deviations.

* *P* < 0.05

** *P* < 0.01, vs. CTR group by Student’s *t*-test.

## Discussion

In the present study, the IMF suppressed the reduction of body weight, food intake, and leukocyte count at early time points after 5-FU injection in rats. The feeding of the IMF also delayed the incidence of diarrhoea with the preservation of the intestinal villi.

One of the strengths of this study is that it presents a possibility of nutritional treatment as a supportive care for the side effects of chemotherapy. Weight loss occurs in 30% to more than 80% of patients with cancer and it is severe (>10% of weight loss) in some cases at the time of diagnosis [16]. Moreover, the adverse effects of cancer chemotherapy influence the nutritional status and body weights of patients receiving chemotherapy [17, 18]. Weight loss also affects the outcome of therapy and is a key determinant of a patient’s quality of life [2]. This study showed that the IMF could delay the incidence of the adverse effects induced by 5-FU, which implies that the nutritional treatment with the IMF might have reduced the deterioration of the nutritional status and contribute beneficially to the quality of life of patients with cancer. Currently, symptomatic treatment with medication is often experienced as the side effects of anticancer drugs [19, 20]. Nutritional therapy using the IMF is also expected to have a dose-sparing effect on medicine for symptomatic treatment.

In this study, the IMF also delayed the incidence of diarrhoea and alleviated intestinal injury. Our previous studies showed that the IMF promoted the growth of villi in the intestinal mucosa [21]. Therefore, the promotion of villi by the nutritional treatment with the IMF leads to the strengthening of the intestinal mucosal barrier, which might enhance the resistance to intestinal toxicity of 5-FU and delay the incidence of diarrhoea. Diarrhoea and leukopenia cause dose limiting of anticancer drugs. The IMF contributes to the completion of chemotherapy and overcoming cancer by suppressing diarrhoea and leukopenia.

One of the limitations of this study is that the contributing factors of the IMF are not clarified. One possible mechanism is the anti-inflammatory effects of the IMF. The injection of 5-FU has been shown to induce pro-inflammatory cytokines and NF-kappaB activation [4, 5]. An anti-inflammatory drug (5-aminosalicylic acid) has been shown to improve 5-FU-induced intestinal injury and to inhibit NF-kappaB activation and pro-inflammatory cytokine production [4]. The IMF used in this study has also been shown to regulate acute and chronic inflammation and to suppress the increase in intestinal permeability and bacterial translocation in some experimental models [9–13]. Therefore, nutrients with anti-inflammatory properties, whey-hydrolysed peptide, omega-3 polyunsaturated fatty acids, and anti-oxidant molecules (vitamin A, C, E, zinc, and selenium), may contribute to the reduction of the side effects of anticancer drugs.

The gut microbiota has been shown to be actively involved in the pathological process of 5-FU-induced intestinal mucositis [22], and probiotics and prebiotics have been reported to modulate the structure of the gastrointestinal tract as well as the composition of microflora, and the immune system [7]. In fact, some probiotics have been shown to reduce the adverse effects of anticancer drugs. The supplementation of *L*. *rhamnosus* GG has been reported to reduce diarrhoea and abdominal discomfort and to lessen the dose limit of anticancer drugs in colorectal cancer patients receiving 5-FU [7, 23]. Another Lactobacillus reduced anorexia and body weight loss induced by 5-FU [24]. It has also been reported that heat-killed yogurt containing *L*. *burgaricus* and *S*. *thermophiles* modulated intestinal microbiota, where useful bacteria such as lactic acid bacteria were increased [25]. In our previous study, the IMF also increased the numbers of *Bifidobacterium* and *Lactobacillus* in the cecum of rats as well as promoted the growth of villi in the intestinal mucosa [21]. Therefore, the fermented milk contained in the IMF seems to contribute partly to preventing the adverse effects of 5-FU, because the fermented milk in the IMF also contains *L*. *burgaricus* and *S*. *thermophiles*.

Another limitation of this study is that we did not investigate whether the IMF affects the antitumor effects of drugs. One possible mechanism by which the IMF prevented the adverse effects of 5-FU is by attenuating the chemotherapeutic efficacy. However, we demonstrated in the previous study that the IMF, in combination with chemotherapy, alleviated cancer cachexia without suppressing chemotherapeutic efficacy in mice [13]. Therefore, we do not believe that our present results are accompanied with weakening of the anti-cancer effect of chemotherapy drugs. Further, the effectiveness and effective dose of the IMF in humans were not confirmed in this study, which is also a limitation. Further clinical trials are required to establish the efficacy and safety of the IMF for cancer patients. Although we recognize that further studies are needed to determine the mechanism by which the IMF preserved body weight, food intake, leukocyte count, and intestinal villi, and to confirm these effect in humans, we believe that nutritional treatment with the IMF during cancer chemotherapy could be a new supportive therapy for cancer patients.

## Supporting information

S1 Table Nutritional contents of the IMF (per 100 kcal). (DOCX)

S1 Dataset Raw dataset. (XLSX)

## References

[1] Prado CM, Lieffers JR, McCargar LJ, Reiman T, Sawyer MB, Martin L, et al Prevalence and clinical implications of sarcopenic obesity in patients with solid tumours of the respiratory and gastrointestinal tracts: a population-based study. Lancet Oncol. 2008; 9(7): 629–635. Epub 2008/06/10. 10.1016/S1470-2045(08)70153-0 18539529

[2] Ravasco P, Monteiro-Grillo I, Vidal PM, Camilo ME. Cancer: disease and nutrition are key determinants of patients' quality of life. Support Care Cancer. 2004; 12(4): 246–252. Epub 2004/03/05. 10.1007/s00520-003-0568-z 14997369

[3] Stringer AM, Gibson RJ, Logan RM, Bowen JM, Yeoh AS, Hamilton J, et al Gastrointestinal microflora and mucins may play a critical role in the development of 5-Fluorouracil-induced gastrointestinal mucositis. Exp Biol Med (Maywood). 2009; 234(4): 430–441. Epub 2009/01/30. 10.3181/0810-rm-301 19176868

[4] Chang CT, Ho TY, Lin H, Liang JA, Huang HC, Li CC, et al 5-Fluorouracil induced intestinal mucositis via nuclear factor-kappaB activation by transcriptomic analysis and in vivo bioluminescence imaging. PLoS One. 2012; 7(3): e31808 Epub 2012/03/14. 10.1371/journal.pone.0031808 22412841 PMC3296709

[5] Logan RM, Stringer AM, Bowen JM, Gibson RJ, Sonis ST, Keefe DM. Serum levels of NFkappaB and pro-inflammatory cytokines following administration of mucotoxic drugs. Cancer Biol Ther. 2008; 7(7): 1139–1145. Epub 2008/06/07. 10.4161/cbt.7.7.6207 18535404

[6] Masuda Y, Inoue M, Miyata A, Mizuno S, Nanba H. Maitake beta-glucan enhances therapeutic effect and reduces myelosupression and nephrotoxicity of cisplatin in mice. Int Immunopharmacol. 2009; 9(5): 620–626. Epub 2009/03/03. 10.1016/j.intimp.2009.02.005 19249389

[7] Xue H, Sawyer MB, Wischmeyer PE, Baracos VE. Nutrition modulation of gastrointestinal toxicity related to cancer chemotherapy: from preclinical findings to clinical strategy. JPEN J Parenter Enteral Nutr. 2011; 35(1): 74–90. Epub 2011/01/13. 10.1177/0148607110377338 21224434

[8] Pontes-Arruda A, Demichele S, Seth A, Singer P. The use of an inflammation-modulating diet in patients with acute lung injury or acute respiratory distress syndrome: a meta-analysis of outcome data. JPEN J Parenter Enteral Nutr. 2008; 32(6): 596–605. Epub 2008/11/01. 10.1177/0148607108324203 18974237

[9] Kume H, Okazaki K, Takahashi T, Yamaji T. Protective effect of an immune-modulating diet comprising whey peptides and fermented milk products on indomethacin-induced small-bowel disorders in rats. Clin Nutr. 2014; 33(6): 1140–1146. Epub 2014/01/28. 10.1016/j.clnu.2013.12.014 24461940

[10] Kume H, Okazaki K, Yamaji T, Sasaki H. A newly designed enteral formula containing whey peptides and fermented milk product protects mice against concanavalin A-induced hepatitis by suppressing overproduction of inflammatory cytokines. Clin Nutr. 2012; 31(2): 283–289. Epub 2011/11/29. 10.1016/j.clnu.2011.10.012 22119211

[11] Nakamura K, Fukatsu K, Sasayama A, Yamaji T. An immune-modulating formula comprising whey peptides and fermented milk improves inflammation-related remote organ injuries in diet-induced acute pancreatitis in mice. Biosci Microbiota Food Health. 2018; 37(1): 1–8. Epub 2018/02/02. 10.12938/bmfh.17-011 29387516 PMC5787410

[12] Nakamura K, Ogawa S, Dairiki K, Fukatsu K, Sasaki H, Kaneko T, et al A new immune-modulating diet enriched with whey-hydrolyzed peptide, fermented milk, and isomaltulose attenuates gut ischemia-reperfusion injury in mice. Clin Nutr. 2011; 30(4): 513–516. Epub 2011/02/02. 10.1016/j.clnu.2011.01.002 21281994

[13] Nakamura K, Sasayama A, Takahashi T, Yamaji T. An immune-modulating diet in combination with chemotherapy prevents cancer cachexia by attenuating systemic inflammation in colon 26 tumor-bearing mice. Nutr Cancer. 2015; 67(6): 912–920. Epub 2015/07/03. 10.1080/01635581.2015.1053495 26133950

[14] Trifan OC, Durham WF, Salazar VS, Horton J, Levine BD, Zweifel BS, et al Cyclooxygenase-2 inhibition with celecoxib enhances antitumor efficacy and reduces diarrhea side effect of CPT-11. Cancer Res. 2002; 62(20): 5778–5784. Epub 2002/10/18. 12384538

[15] Xue H, Sawyer MB, Field CJ, Dieleman LA, Baracos VE. Nutritional modulation of antitumor efficacy and diarrhea toxicity related to irinotecan chemotherapy in rats bearing the ward colon tumor. Clin Cancer Res. 2007; 13(23): 7146–7154. Epub 2007/12/07. 10.1158/1078-0432.CCR-07-0823 18056195

[16] Arends J, Bodoky G, Bozzetti F, Fearon K, Muscaritoli M, Selga G, et al ESPEN Guidelines on Enteral Nutrition: Non-surgical oncology. Clin Nutr. 2006; 25(2): 245–259. Epub 2006/05/16. 10.1016/j.clnu.2006.01.020 16697500

[17] Yang YH, Lee DS. The relationship of anorexia, nausea, vomiting, oral intake and nutritional status in patients receiving chemotherapy. J Korean Acad Nurs. 2000; 30(3): 720–730.

[18] Lee H-O, Lee J-J. Nutritional intervention using nutrition care process in a malnourished patient with chemotherapy side effects. Clin Nutr Res. 2015; 4(1): 63–67. Epub 12/08. 10.7762/cnr.2015.4.1.63 25713794 PMC4337925

[19] Cinausero M, Aprile G, Ermacora P, Basile D, Vitale MG, Fanotto V, et al New frontiers in the pathobiology and treatment of cancer regimen-related mucosal injury. Front Pharmacol. 2017; 8(354). 10.3389/fphar.2017.00354 28642709 PMC5462992

[20] Nurgali K, Jagoe RT, Abalo R. Editorial: adverse effects of cancer chemotherapy: Anything new to improve tolerance and reduce sequelae? Front Pharmacol. 2018; 9: 245–245. 10.3389/fphar.2018.00245 29623040 PMC5874321

[21] Kume H, Nakamura K, Okazaki K, Matsuura M, Yamaji T, Ashida K. Influence of nutritional management using an enteral formula MHN-02 on intestinal tissue structure in rats. Milk Sci. 2018; 67(1): 30–33. 10.11465/milk.67.30

[22] Li H-L, Lu L, Wang X-S, Qin L-Y, Wang P, Qiu S-P, et al Alteration of gut microbiota and inflammatory cytokine/chemokine profiles in 5-fluorouracil induced intestinal mucositis. Front Cell Infect Microbiol. 2017; 7(455). 10.3389/fcimb.2017.00455 29124041 PMC5662589

[23] Osterlund P, Ruotsalainen T, Korpela R, Saxelin M, Ollus A, Valta P, et al Lactobacillus supplementation for diarrhoea related to chemotherapy of colorectal cancer: a randomised study. Br J Cancer. 2007; 97(8): 1028–1034. Epub 2007/09/27. 10.1038/sj.bjc.6603990 17895895 PMC2360429

[24] Von Bultzingslowen I, Adlerberth I, Wold AE, Dahlen G, Jontell M. Oral and intestinal microflora in 5-fluorouracil treated rats, translocation to cervical and mesenteric lymph nodes and effects of probiotic bacteria. Oral Microbiol Immunol. 2003; 18(5): 278–284. Epub 2003/08/22. 10.1034/j.1399-302x.2003.00075.x 12930518

[25] Garcia-Albiach R, Pozuelo de Felipe MJ, Angulo S, Morosini MI, Bravo D, Baquero F, et al Molecular analysis of yogurt containing Lactobacillus delbrueckii subsp. bulgaricus and Streptococcus thermophilus in human intestinal microbiota. Am J Clin Nutr. 2008; 87(1): 91–96. Epub 2008/01/08. 10.1093/ajcn/87.1.91 18175741

