# Supplementary material for: Nutritional treatment with an immune-modulating enteral formula alleviates 5-fluorouracil-induced adverse effects in rats
Source: PLoS One. 2019 Nov 26;14(11):e0225389. doi: 10.1371/journal.pone.0225389 (PMC6879153; doi:10.1371/journal.pone.0225389)
Supplement: S1 Table — (DOCX) [file pone.0225389.s001.docx]

**S1 Table. Nutritional contents of the IMF (per 100 kcal).**

|  | IMF |
| --- | --- |
| Protein (g) | 5.0 |
| Protein sources | Whey-hydrolysed peptides,  fermented milk |
| Carbohydrates (g) | 14.5 |
| Carbohydrate sources | Dextrin,  Isomaltulose |
| Lipids (g) | 2.8 |
| Lipid sources | LCT, MCT,  EPA, DHA |
| *Vitamins* |  |
| Vitamin A ($\mu$g RE ^e^) | 150 |
| Vitamin D ($\mu$g) | 0.75 |
| Vitamin E (mg) | 5.0 |
| Vitamin K ($\mu$g) | 3.4 |
| Vitamin B1 (mg) | 0.25 |
| Vitamin B2 (mg) | 0.30 |
| Niacin (mg) | 3.0 |
| Vitamin B6 (mg) | 0.30 |
| Vitamin B12 ($\mu$g) | 0.60 |
| Folic acid ($\mu$g) | 50 |
| Biotin ($\mu$g) | 7.5 |
| Vitamin C (mg) | 50 |
| Choline (mg) | 9.2 |
| *Minerals* |  |
| Sodium (mg) | 70 |
| Potassium (mg) | 80 |
| Calcium (mg) | 80 |
| Magnesium (mg) | 20 |
| Phosphorus (mg) | 70 |
| Iron (mg) | 1.0 |
| Zinc (mg) | 1.0 |
| Copper (mg) | 0.050 |
| Manganese (mg) | 0.18 |
| Chromium ($\mu$g) | 3.0 |
| Molybdenum ($\mu$g) | 2.5 |
| Selenium ($\mu$g) | 5.0 |
| Iodine ($\mu$g) | 9.7 |
| Chloride (mg) | 80 |

^a^LCT, long chain triglycerides

^b^MCT, medium chain triglycerides

^c^EPA, eicosapentaenoic acid

^d^DHA, docosahexaenoic acid

^e^RE, retinol equivalent
